# Supplementary material for: Causal Relationship between Aging and Anorexia Nervosa: A White-Matter-Microstructure-Mediated Mendelian Randomization Analysis
Source: Biomedicines. 2024 Aug 16;12(8):1874. doi: 10.3390/biomedicines12081874 (PMC11351342; doi:10.3390/biomedicines12081874)
Supplement: Supplementary file 1 [file biomedicines-12-01874-s001.zip › Supplementary Figure/Figure S8/Leaveoneout plot of MR analysis for TL to ubm-b-1702.pdf]

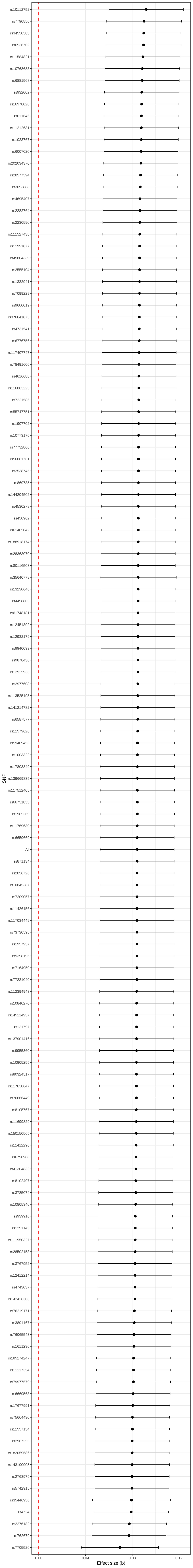

rs10112752
rs7790056
rs34550383
rs6536702
rs11584821
rs10768683
rs6881568
rs932002
rs16978028
rs611646
rs11212631
rs1023767
rs6007020
rs202034370
rs28577594
rs3093888
rs4695407
rs22282764
rs2230590
rs111527438
rs11991877
rs45604339
rs2555104
rs1332941
rs7099229
rs9600019
rs376641875
rs4731541
rs6776756
rs117407747
rs78491606
rs4616688
rs116863223
rs7221585
rs55747751
rs1907702
rs10773176
rs77732866
rs56061761
rs2538745
rs869785
rs144204502
rs4530278
rs450962
rs61405042
rs188918174
rs28363070
rs80116508
rs35640778
rs13230646
rs4498805
rs61748181
rs12451892
rs12932179
rs9940099
rs9878436
rs12925933
rs2977608
rs113525195
rs141214782
rs6587577
rs11579626
rs59409453
rs1003322
rs17803849
rs139669835
rs117512405
rs66731853
rs1985369
rs11769630
rs6659669
A8
rs871134
rs2056726
rs10845387
rs7209057
rs11426156
rs117034449
rs73730598
rs1957937
rs9398196
rs7164950
rs77231040
rs112394943
rs10840270
rs145114957
rs131797
rs137901416
rs9955360
rs10905255
rs80324517
rs117630647
rs76866449
rs8105767
rs11699829
rs150150565
rs11412296
rs6790988
rs41304832
rs8102497
rs3785074
rs10805346
rs939916
rs1291143
rs111950327
rs28502153
rs3767952
rs12412214
rs4743037
rs142426306
rs76219171
rs3891167
rs76065543
rs1611236
rs185174247
rs11117354
rs79977579
rs6669563
rs17677991
rs75664430
rs11557154
rs2967355
rs182059586
rs143190905
rs2763979
rs57742915
rs35446936
rs4724
rs2276182
rs762679
rs7705526
SNP
0.00
0.04
0.08
0.12
Effect size (b)
